# FDG-avid pulse granulomas mimicking peritoneal metastases in colorectal cancer: a case report

**DOI:** 10.1093/jscr/rjag826

**Published:** 2026-09-18

**Authors:** Susanna Li, Cameron Law

**Affiliations:** School of Clinical Medicine, UNSW Sydney, Randwick, NSW, Australia; Department of Colorectal Surgery, Prince of Wales Hospital, Randwick, NSW, Australia; Department of Colorectal Surgery, Prince of Wales Hospital, Randwick, NSW, Australia

**Keywords:** pulse granulomas, foreign body granuloma, colorectal cancer, peritoneal metastases, case report

## Abstract

Pulse granulomas are a rare, benign foreign body granulomatous reaction, characterized by hyaline rings, surrounded by multinucleated cells. While typically reported in the oral cavity and lungs, reports on extraoral gastrointestinal manifestations are limited. We present a case on a 72-year-old gentleman with a history of bowel perforation who was found to have a splenic flexure adenocarcinoma on surveillance colonoscopy. Staging imaging showed innumerable fluorodeoxyglucose-avid peritoneal nodules, with concerns of metastatic disease. Diagnostic laparoscopy and peritoneal biopsy found pulse granulomas. The patient had a left hemicolectomy and a final staging of T3N1M0 adenocarcinoma without distant metastases. This case demonstrates pulse granulomas as a rare but relevant mimic of peritoneal metastatic disease in colorectal cancer. Pulse granulomas should be a differential diagnosis of peritoneal nodules in patients with previous mucosal injury. Histopathological confirmation must be pursued to prevent unnecessary surgical intervention or excluding patients from curative treatment.

## Introduction

Pulse granulomas are a rare, benign foreign body granulomatous reaction. It has pathognomonic histological findings of hyaline rings circumscribed by multinucleated giant cells and histiocytes [1]. The predominant theory behind its pathogenesis posits that hyaline rings arise from cellulose secondary to degraded vegetable material [2]. Pulse granulomas are typically found in the oral cavity and lungs from implanted and aspirated food particles [3]. There is limited data on pulse granulomas in extraoral gastrointestinal settings. There is only one case series to date reporting on 22 resected gastrointestinal specimens with pulse granulomas from 17 patients. All patients reported a history of intestinal injury, including perforation and diverticular disease. Most specimens were nodular and multifocal with the colorectum and small intestine being commonly involved; only 1/22 specimens involved the peritoneum. About 7/22 specimens had imaging and intraoperative findings concerning for malignancy [4].

The authors present an interesting case where a patient with colorectal cancer had diffuse pulse granulomas, masquerading as metastatic peritoneal disease.

## Case report

The patient is a 72-year-old gentleman with a complicated abdominal surgical history. He is an independent man from home with a background of obstructive sleep apnoea, atrial fibrillation, provoked deep venous thrombosis, non-smoker, and has a body mass index of 37.

In 2024, he had a laparotomy and small bowel resection for an incarcerated ventral hernia, requiring laparostomy, and necessitating a take back for closure Day 1 post-operatively due to significant intraoperative inotropic requirement. He underwent a further resection in 2025 for small bowel obstruction and perforation, secondary to a recurrent ventral hernia. His post-operative recovery was complicated by polymicrobial abdominal collections.

In March 2026, he underwent a gastroscopy and colonoscopy for gastric ulcer surveillance and iron deficiency anaemia. This found a semi-circumferential ulcerated lesion at the splenic flexure. The patient denied any obstructive symptoms or rectal bleeding in the preceding months. His carcinoembryonic antigen (CEA) level was normal (<2 μg/L).

The patient underwent staging imaging. Computed tomography (CT) findings showed multiple peritoneal nodules scattered in the anterior abdominal wall, left paracolic gutter and left hemipelvis, as well as small right upper lobe and lateral lingual lung nodules. His fluorodeoxyglucose–positron emission tomography (FDG-PET) scan demonstrated a metabolically active lesion in the splenic flexure [standardized uptake value (SUV) maximum 17.3] but no nodal disease. Of interest, there were innumerable moderately to intensively metabolically active peritoneal nodules (SUV maximum 9.1) corresponding to those seen on CT. These were concerning for disseminated peritoneal disease.

**Figure 1:**
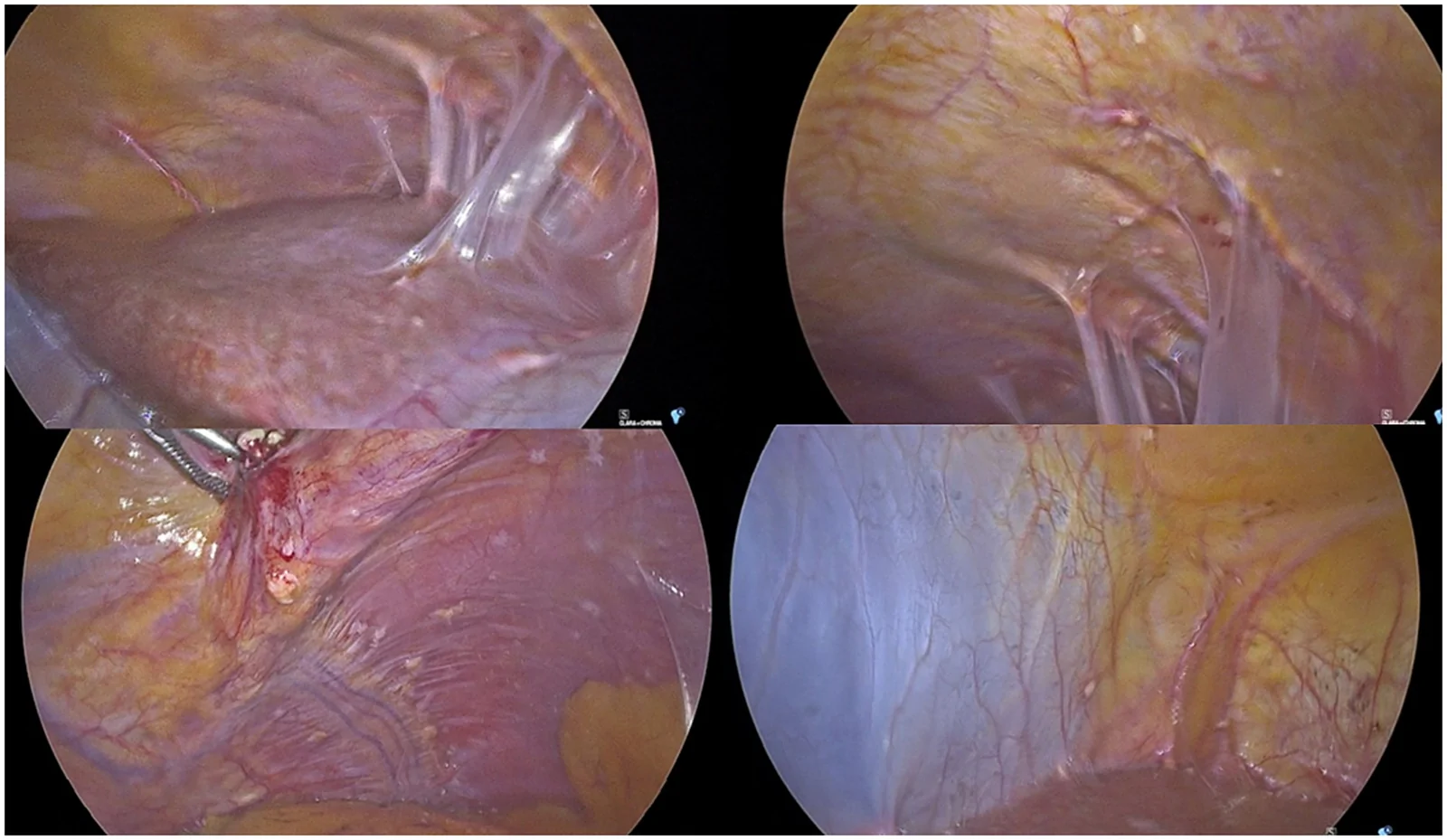
Laparascopic views of extensive peritoneal nodules in the upper abdomen.

He was originally planned for a laparoscopic division of adhesions and left hemicolectomy. Considering these PET findings however, he ultimately underwent a diagnostic laparoscopy, omental and peritoneal biopsy and flexible sigmoidoscopy in March 2026. Intraoperative findings included extensive adhesions from previous surgeries and multiple subcentimetre peritoneal nodules (Fig. 1). Due to the extent of adhesions, a peritoneal cancer index was unable to be accurately determined. Peritoneal nodules and sigmoid colon samples were sent for urgent histopathology.

The histopathology results were striking. Peritoneal nodule specimens showed pulse granulomas consisting of multinucleated giant cell reaction to vegetable matter with no histological evidence of malignancy. As expected, the splenic flexure sample confirmed the presence of an adenocarcinoma.

In May 2026, the patient ultimately underwent a laparotomy, adhesiolysis, incisional hernia repair, and left hemicolectomy with a side-to-side stapled anastomosis. Histopathology confirmed the presence of multiple mesenteric pulse granulomas, likely in the context of previous small bowel perforations. The final staging of the splenic flexure tumour adenocarcinoma was T3N1M0R0, notably without distant metastases as previously suspected.

The patient recovered well and returned to his pre-operative level of function, with multidisciplinary recommendations for adjuvant chemotherapy.

## Discussion

Foreign body granulomas caused by surgical sutures, sponges and post-chemotherapy activated charcoal have been reported as a mimic of malignancy, resulting in unnecessary surgical intervention [5–8]. Data on pulse granulomas remains limited.

To our knowledge, this is one of few cases describing pulse granulomas with positive pre-operative FDG-PET avidity mimicking peritoneal metastasis in colorectal cancer. The presence of peritoneal pulse granulomas mimicking metastases from a primary colorectal cancer influenced initial clinical management. Peritoneal involvement indicates stage IV colon cancer which is potentially non-resectable compared to stage III which is typically managed with surgical resection and adjuvant chemotherapy [9]. Pulse granulomas are a rare but important benign differential of peritoneal metastases as misclassification can substantially alter and/or delay treatment planning.

Prior mucosal injury is present in nearly all reported cases [4, 7, 8, 10, 11]. This is consistent with our patient with a history of multiple small bowel perforations. Patients with previous mucosal injury from medical conditions or surgical intervention are at increased risk of pulse granulomas.

CEA levels have limited sensitivity in early-stage colorectal cancer. Sensitivity rises from 38.1% in stage 1 to 78.3% in stage IV disease with 90% specificity [12]. With a consideration of clinical history and imaging, discordant biochemistry findings in patients with suspected peritoneal metastatic disease should warrant consideration of other differentials, such as pulse granulomas.

Imaging is overall unreliable, with a recent report of normal pre-operative CT and FDG-PET scans in a patient undergoing colorectal cancer resection. Macroscopic peritoneal nodules (later histologically proven to be foreign body granulomas) were discovered intraoperatively, resulting in an abandoned resection [13]. CT findings in pulse granulomas have been reported as lesions with central hypointensity suggestive of necrosis, which are nonspecific and easily mistaken for malignancy [7]. Positive FDG-PET findings have been reported in foreign body granulomas involving surgical material or post-chemotherapy charcoal [14, 15]. FDG-PET avidity varies with foreign material type and associated peripheral inflammation [11].

Histopathology is the only definitive method to distinguish benign pulse granulomas from peritoneal malignancy. As demonstrated, FDG-PET and CT morphology does not reliably exclude benign causes. In cases of colorectal cancer, where peritoneal disease is identified on imaging or intraoperatively, tissue sampling must be pursued to ensure accurate staging and management.

## Conclusion

Although rare, a high index of suspicion for pulse granulomas should be maintained in patients with prior mucosal injury as there is an almost universal association [4]. When clinical, biochemical and imaging findings are conflicting, the possibility of a benign pulse granuloma should be considered before attributing peritoneal findings to metastatic colorectal disease to avoid unnecessary surgical intervention or exclusion from curative management.
